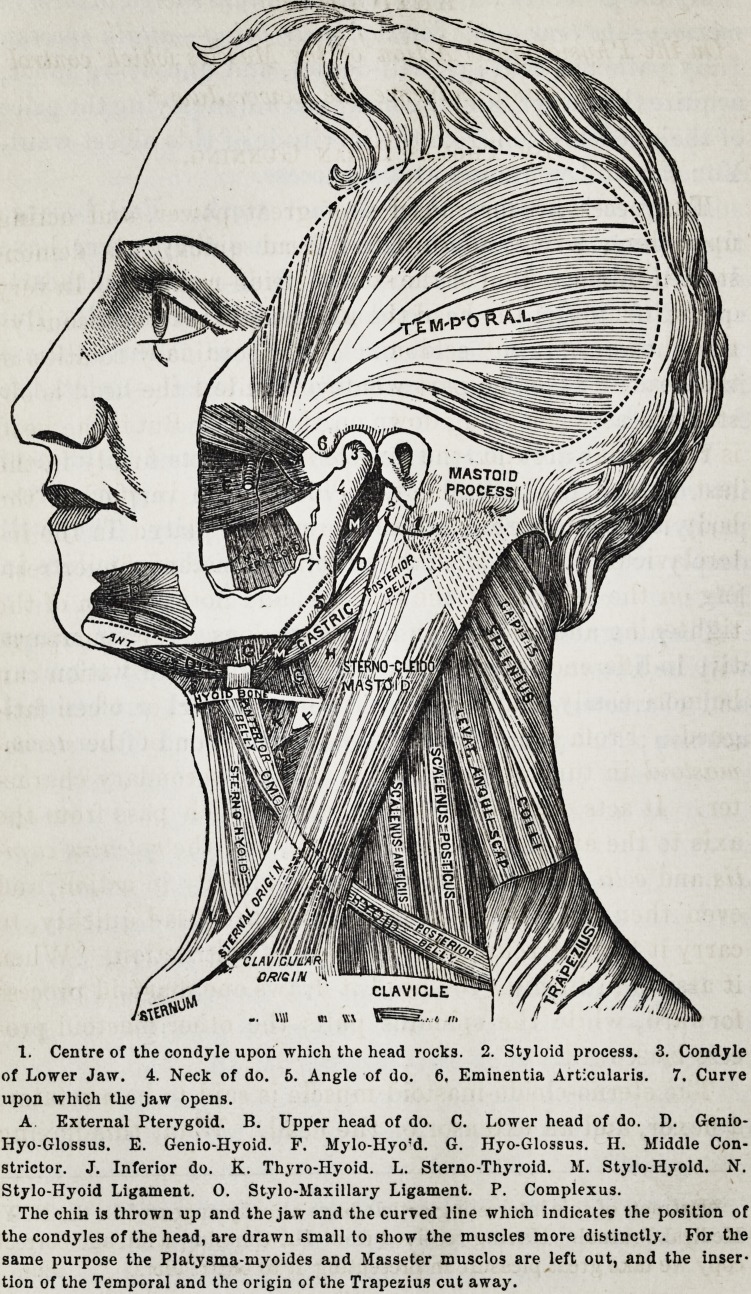# On the Physiological Action of the Muscles Which Control and Influence the Lower Jaw


**Published:** 1868-04

**Authors:** Thomas Brian Gunning


1. Centre of the condyle upon which the head rocks. 2. Styloid process. 3. Condyle of Lower Jaw. 4. Neck of do. 5. Angle of do. 6. Eminentia Articularis. 7. Curve upon which the jaw opens.

A. External Pterygoid. B. Upper head of do. C. Lower head of do. D. Genio-Hyo-Glossus. E. Genio-Hyoid. F. Mylo-Hyoid. G. Hyo-Glossus. H. Middle Constrictor. J. Inferior do. K. Thyro-Hyoid. L. Sterno-Thyroid. M. Stylo-Hyoid. N. Stylo-Hyoid Ligament. O. Stylo-Maxillary Ligament. P. Complexus.

The chin is thrown up and the jaw and the curved line which indicates the position of the condyles of the head, are drawn small to show the muscles more distinctly. For the same purpose the Platysma-myoides and Masseter musclos are left out, and the insertion of the Temporal and the origin of the Trapezius cut away.

### ARTICLE II.

### *On the Physiological Action of the Muscles which control and influence the Lower Jaw.**

By Thomas Brian Gunning.

The necessity for muscles of great power, and acting upon long levers, to turn the head quickly, is demonstrated by the action of the sterno-cleido-mastoid. In very quick turning of the head the muscle acts instantaneously; this, however is but seldom. In the ordinary rotation of the head, it takes no part whatever, unless the head is obstructed as when lying down on the side. But if the head is turned far around, the muscle always acts firmly in the last part of the movement. This can be verified if the body is held upright and the forefinger placed in the interclavicular notch, with the thumb and second finger resting on the tendons of the muscles, and notice taken of the tightening and relaxation of the tendons. The comparative indifference of this muscle to the head's rotation can be more easily demonstrated in the evening, or when fatigued. From this it appears that the action of the *sterno-mastoid* in turning the head is of very secondary character. It acts only when the rotators which pass from the axis to the atlas and occipital bone, and the *splenius capitis* and *colli* of the opposite side, are already in action, and even then only to assist in turning the head quickly, to carry it further round, or to overcome obstruction. When it assists in turning the head it draws one mastoid process forward, while the splenius pulls the other mastoid process backward.

The sterno-cleido-mastoid muscle is said to be a rotator, a flexor, and an extensor of the head. What this flexing

---

*Extracts of this interesting article have already appeared in the N. Y. Medical Journal. Having received from Dr. Gunning a full and correct copy we take great pleasure in presenting it to our readers. *Eds.*

of the head means, in addition to lateral movement, may be learned by the following quotation. "The sterno-mastoid muscles, when both are brought into action, serve to depress the head upon the neck, and the neck upon the chest."* These views are also maintained by J. Cruveilhier† and may be accepted as those not only of the French anatomists and physiologists generally, but also of the German and English with few exceptions. Professor Henle, however, says positively that these muscles do not flex the head down in front, and that they lift the head and bend the neck when the body is brought up in rising from the back.‡ This is a great advance upon what is said by others, but beyond this he gives no intimation of understanding their peculiar and most important function.

The insertion of the sterno-cleido-mastoid muscle is around the front of the mastoid process, and back along the superior curve line, about half the distance between the mastoid process and the centre of the occipital protuberance, while the front of the mastoid process is nearly always on a line with the centre of the condyles of the occipital bone (in rare instances, however, it is nearer the front of the condyle). The sterno-cleido-mastoid muscle is consequently inserted back of the centre upon which the head rocks (except in rare cases when a small portion of the muscle is a little forward of it). Notwithstanding this, it is set down as rocking the head forward, and the action of the muscle in rising is brought forward to prove it. This experiment, however, if properly conducted and explained, will prove the contrary. If the experimenter, while lying flat on his back, with the forefinger resting in the interclavicular notch, and the thumb and second finger on the tendons, will raise his head and shoulders a little, he will find that the muscles are acting strongly ; then

* Gray's Anatomy. 2d Amer. edit., p. 256. Phil. 1865.

† Traite d'Anatomie Descriptive. Troisieme edit., tome deuxieme, p 173. Paris. 1851.

‡ Handbuch der Muskellehre des Menschen von Dr. T. Henle, Professor der Anatomie in Goettingen. (Pege 110).

by staying in that position and rocking the head backward and forward, it will be felt that the muscles are unaffected in any part of their fibres, and that they pay no attention to the movement of the head, neither the tendons on the sternal portions nor those on the clavicular being relaxed for a moment. Then sit up, throw the head forward sufficiently to relax the tendons, and rock the head as before; it will now be found that the tendons remain relaxed, showing that tightness of the tendons did not conceal action of the muscles in the first experiment, and demonstrating that the *sterno-cleido-mastoid* muscles do *not* "serve to depress the head upon the neck." In bringing the head forward these muscles act only until the head comes to its centre of balance, when the tendons relax and remain so, even when the chin touches the breast. But if the head is obstructed in this downward movement, these muscles will then assist to bring it down in front and hold it there. The sterno-cleido-mastoid muscles do not, however, in this rock the head upon the atlas, but bring and keep the atlas forward. Neither are they "extensors of the head" in the sense indicated by the books, which seems at first sight to accord more with their insertion back of the centre of the condyles. But the insertion is so peculiar that it requires consideration to determine how the muscles affect the head. The mastoid process is always below the superior curved line upon which the back part of the muscle is inserted. When the process is large it may be more than an inch below it, although much less when the process is small, as in childhood before the cells are developed. Moreover, the uniformity of position between the mastoid processes and the condyles horizontally is not met with in their vertical relation, the condyles being on some skulls more than half an inch lower than the mastoid processes, while on others the processes are as much below the condyles, the large proportion being between these extremes. These variations go far to show that the sterno-mastoid muscles are not intended to rock the head backward, for when the mastoid process is much lower than the condyles, and especially when it is large and projects forward somewhat, to correspond to the direction of the muscle it follows that as the head is pulled downward (by the *trapezii*, &c.) the mastoid processes go upward and forward; consequently if the *sterno-cleido-mastoid* were to act to bring the head down behind, the portion on the mastoid process—the strongest part of the muscle—would hold the head down in front, probably as much as that on the occipital bone would pull it down behind. But their action can be tested by lying down so as to remove the necessity for action of the muscles to hold the atlas. In this position (care being taken not to lift the atlas, or neck) the *sternal* portion of the muscles will not act in concert with the other muscles to rock the head back, even if the whole weight of the body is thrown upon the back of the head, and I have been unable to find any action in the clavicular portion, although the action of this part of the muscle is so delicate and prompt that it can be distinctly felt when the foot is raised in walking, the head and body being then thrown over to the other side to restore the balance. Further, when the *sterno-cleido-mastoid* and the *splenius* of the same side are acting in concert to pull the head down to the shoulder, no backward movement of the head is discoverable. *This* is conclusive, for both these muscles having similar insertions, if one rocks the head back the other must, and their combined action would be manifest if they exerted it.

It has been previously shown that this muscle acts as a *rotator* only by sometimes assisting the *splenius*, &c., of the opposite side, and as a lateral *flexor*, in connection with the *splenius* of the same side, but only when the head is obstructed, and then generally by its clavicular portion, the sternal acting only in extreme necessity. It is now seen that it does not flex the head down in *front*, that is upon the atlas, and that its action as an *extensor* of the head can not be demonstrated. The proper function of the

*sterno-cleido-mastoids* when acting in concert, is to give anterior support to the top of the spine, the *splenii* muscles giving posterior support. This may be easily proved by sitting down and watching the tendons. When the head is back of its centre of support both the sternal and clavicular tendons are tightened, when rising they become tenser until the head is started, as it comes into balance they relax. On sitting down, the tendons tighten to check the head as it goes back out of balance. Sudden forward movements tighten them until the head is in motion, they then slacken as the head is forward of the centre and the atlas supported by the *splenii* muscles. If the head is in balance, any pressure upon the forehead acts with increased force upon the atlas and brings the muscles into action to keep it upright. The action of the sterno-cleido-mastoid muscles in these movements is but a modification of the service rendered by them in raising the head from the horizontal position, in doing which the muscles at first support more than the weight of the head, for in supporting the mastoid processes they support the atlas, and make it a fulcrum between the bulk of the head and the counterbalance at the other end of the lever, but as the body comes upright and the head into balance, the strain upon the sterno-mastoid muscles gradually diminishes, until the head is held by the posterior muscles, when the atlas bears all the weight *vertically*.

[A reference to the figure will render this explanation more apparent. The same figure also illustrates the action of the muscles of the lower jaw, and confirms the opinions expressed in the subsequent portions of this paper.]

The hyoid bone, in addition to the muscles which pass to it from parts above the lower border of the jaw, gives attachment to others, which pass up the front of the neck below the jaw. Of these the *sterno-thyroid* arises close to the centre of the posterior surface of the upper bone of the sternum, and falling back somewhat as it passes up, is inserted into the side of the thyroid cartilage, from whence the *thyro-hyoid* (appearing like a continuation of the preceding) goes up and is inserted into the body and greater cornu of the hyoid bone. The *sterno-hyoid* arises from the sternum and end of the clavicle and is inserted into the lower border of the body of the hyoid bone. It is separated considerably from its fellow at its origin, but crosses the sterno-thyroid and approaches it in the middle of its course; it leaves the front of the thyroid cartilege uncovered.

The *omo-hyoid* arises from the upper border of the scapula, and occasionally from the transverse ligament which crosses the supra-scapular notch. It passes across and up the side of the neck to be inserted into the body of the hyoid bone. It crosses under the *trapezius* and *sterno-cleido-mastoid* muscles but over the *scaleni* and *thyro-hyoid.* It is a double-bellied muscle united by a tendon which is held down by a process of the deep cervical fascia. The first portion is nearly horizontal in its course, but underneath the sterno-mastoid muscle, where the cervical fascia passes around the tendon, it turns up so that the second portion is nearly vertical in its course to the hyoid bone. These are the directions of the muscle when at rest, but when active it approaches the line of its attachments and the cervical fascia is drawn upward and backward.

The *digastric*, another double-bellied muscle, has peculiar relations with the preceding. It arises from the digastric notch, on the inner side of the mastoid process of the temporal bone, and passes downward, forward, and inward, to the side of the hyoid bone, where its rounded tendon (after passing through the stylo-hyoid muscle) is held by an aponeurotic loop in connection with the side of the body of the hyoid bone above the insertion of the omo-hyoid. The muscle then passes forward and is inserted into a large depression on the inner side of the lower border of the jaw close to the symphysis. The tendon which divides the posterior and longer belly from the anterior, gives off a large aponeurotic layer, which is attached to the body and great cornu of the hyoid bone; and with the portion on the opposite side is termed the *supra-hyoid aponeurosis*, which forms a strong layer of fascia between the anterior portions of the two muscles, and a firm investment for the other muscles of this region. The *digastric* muscle is peculiar in not being inserted into the hyoid bone, but attached to it by a loop; this allows the muscle to act without interfering too much with the hyoid bone. The muscle has not, however, that freedom which is attributed to it as a reflected cord, for its aponeurotic connection with the hyoid bone and adjoining muscles prevents it from sliding through the loop which attaches it to the hyoid bone, except to a very limited extent. This powerful muscle exerts great influence from the various and important movements in which it takes part.

The last muscle to be described in this connection, the *platysma myoides*, is very distinctly separated from all the others. It is a broad thin plane of muscular fibres, immediately beneath the skin, on the side of the neck. It arises from the clavicle and acromion, and from the fascia covering the upper part of the pectorial, deltoid and trapezius muscles, and going upward and forward, it covers in the angle and the border of the jaw to the symphysis. It is inserted in the lower border of the jaw in front, but back of the commissure of the lips it is found interlaced with the muscles above. It affords muscular support to the integument, and cover to the muscles beneath, but leaves the thyroid cartilage and the front of the trachea free.

The service supposed to be rendered by the foregoing muscles is shown by the following selections.

J. Cruveilhier says: "The *sterno-hyoid*, the *omo-hyoid*, the *sterno-thyroid*, and the *thyro-hyoid* are the simplest in their structure and the simplest in their action; all coöperate to the lowering of the jaw. Moreover, if the jaw is fixed, they flex the head."*

---

* Traite d'Anatomie Descriptive. 3me ed., tome 2me, p. 179. Paris, 1851.

Sappey says: "The *genio-hyoid* is the elevator of the hyoid bone when the jaw is fixed; lowerer of the jaw if the hyoid bone is motionless; flexor of the head when the hyoid bone and jaw are both fixed."*

J. Cruveilhier says of the digastric: "If the hyoid bone is fixed the posterior belly becomes the lowerer of the jaw, in consequence of the reflection of the muscle; the anterior and posterior bellies can throw the head backward."†

Jamain says: "If the hyoid bone is fixed, the digastric co-operates in lowering the jaw."‡

Todd's *cyclopædia* says: "When the hyoid bone is fixed by its depressors, and perhaps in some degree retracted by the joint actions of the posterior belly of the digastric and of the omo-hyoid, the anterior belly, both passively as a reflected cord, and actively in virtue of its muscular fibres, depresses the lower jaw, and opens the mouth."||

"Chief action of the omo-hyoids is to tighten the cervical fascia during deglutition; they are also capable of depressing the hyoid bone."¶

Gray's *Anatomy* says of the digastric, mylo-hyoid, and genio-hyoid: "When the hyoid bone is fixed by its depressors and those of the larynx, they depress the lower jaw;"** and further, that in deglutition "the anterior belly of the digastric carries the hyoid bone, &c., upward and forward and the posterior belly upward and backward," and says of the *platysma-myoides*: "Its anterior

---

* Traite d'Anatomie Descriptive. Tome 1, premiere partie, p. 213. Paris, 1850.

† Traite d'Anatomie Descriptive. Troisieme edit., tome deuxieme. p 182. Paris. 1851.

‡ Nouveau Traite Elementaire d'Anatomie Descriptive. p. 180. Paris, 1853.

|| Todd's Cyclopædia. Vol. III., p. 564.

¶ Ibid. p. 563.

** Gray's Anatomy. 2d American Edition, p. 260.

portion, the thickest part of the muscle, depresses the lower jaw."*

Henle thinks: "The platysma-myoides are not depressors of the lower jaw."†

Duchenne says: "Its action being exhausted by the mobility of the integuments of the face, the neck and the chest, it has no longer sufficient strength to depress the lower jaw."‡‡

Cruveilheir says: "The platysmas are sometimes unequal in strength."||||

Ziemssen says: "The muscle is sometimes absent."§§

The absence of the platysma-myoides in some cases, and its inequality in others, proves that it is not of any consequence in depressing the jaw, which is a movement requiring great promptness and exactitude. It may be held between the thumb and finger, near the front of the jaw, and if care is taken to discriminate between it and the integument, it may be felt that the muscle pays no attention to the movement of the jaw.

The muscles which centre in the hyoid bone have power to control and move the organs to which they are attached, (and of which they are in several instances important parts,) subject, however, to the following limitations. The *stylo-hyoid ligament* passes down on each side from the styloid process of the temporal bone to the little horn of the hyoid bone. These ligaments have, therefore, a slanting course, while the *supra-hyoid aponeurotic layer*, between the hyoid bone and the inner side of the front of the jaw, has a horizontal direction. By this arrangement the glottis and its covering, &c., are held at some

---

* Ibid. p. 256.

† Handbuch der Muskellehre des Menschen, von Dr. T Henle, Professor der Anatomie in Goettingen. p. 108.

‡ De l'Electrisation Localisee. p. 380. Paris, 1855.

§§ Traite d'Anatomie Descriptive. Troisieme edit., tome deuxieme. p. 166. Paris, 1851.

|||| Die Electricitæt in der Medicin, p. 44 Berlin, 1857.

distance from the back of the pharynx, and free respiration secured, independent of muscular action, while the hyoid bone can move upward or forward to a considerable extent, and be returned to its natural position when at rest, in any direction that is not back of this resting-place. But below this the downward, and especially the backward movements of the hyoid bone are very limited, being only what is gained by the tightening of the ligaments, &c.

Although a case is sometimes seen in which the *stylo-hyoid* ligaments give place to muscles, in others they are entirely ossified, and the temporal bones and the hyoid bone are in one piece. Showing that, depression of the hyoid below its natural position when at rest, is unnecessary, except to a trifling extent.

The digastric muscle is set down as drawing the hyoid bone backward and forward in deglutition, and as depressing the jaw by acting as "a reflected cord." These services are inconsistent with each other and with the anatomy of the parts. If it were fixed so as to draw the bone backward and forward, it could not slide and be of service as a "reflected cord" sufficiently to the lower jaw. To do the latter, the anterior belly should be inserted higher up the jaw, while a long unrestricted tendon of the muscle should run through a fixed loop on the lower border of the hyoid bone, which last should also be freed from the styloid ligaments, and be drawn down half-way to the sternum every time the jaw opened wide, and proportionally for less opening.

In respect to the united action of both bellies drawing the head backward, it is only necessary to say that the origin of the digastric is partly in front of a line drawn across, just behind the condyles of the occipital bone; it could not, therefore, draw the head back appreciably even if its insertion were directly under its origin. It is consequently a mistake to suppose it can do so when its direction forward is more horizontal than vertical. In fact this muscle is the great agent in drawing the head *forward.* The posterior belly slants down to the hyoid bone, but the anterior is nearly horizontal in its course, and when the muscle acts it tends to the line of its attachments by drawing or endeavoring to draw the hyoid bone upward unless the jaw is much depressed, when, as the muscle is straight, or nearly so, it has no power to raise the hyoid bone. But in several important services the digastric acts in concert with the omo-hyoid. In this way the muscles passing from the hyoid bone to the front of the jaw, including the anterior belly of the digastric, are as effectually antagonized as if a powerful muscle passed from each side of the hyoid bone to the opposite cervical vertebræ, with the advantage of greater length of muscle to contract, and easier adaptation to the movements of the jaw; and the muscles in front of the hyoid bone act, when necessary, in alternation with the omo-hyoid and the posterior belly of the digastric. More frequently, however, the anterior belly of the digastric acts with the posterior belly and the omo-hyoid, for they keep the head upright. In doing this, the omo-hyoid muscle and the posterior belly of the digastric draw or hold the hyoid bone back, while the anterior belly of the digastric brings in the chin, and the *temporal* and other elevators of the jaw draw the head forward; in this way, the digastric acts on a long lever, as the head rocks on a centre, but a little below the entrance of the external ear. The digastric and omo-hyoid muscles are always active during forward or backward movements of the body or head. They do for the head, what the sterno-mastoid muscles do for the spine, and their action can be felt easily with the finger, in sitting down or rising up, &c. They are also powerful rotators of the head, and the action of the omo-hyoid is singularly quick in sudden turnings of the head, (as with the sterno-mastoid muscles,) the digastric being useful in assisting to keep the hyoid bone up in place, it being held laterally by the aponeurosis and probably by the mylo-hyoid muscle.

If the end of the finger is placed just behind the origin of the *cleido-mastoid* during these movements, the omo-hyoid will be felt rising above the clavicle, and carrying the cervical fascia upward and backward; and if a finger is placed behind the mastoid process so as to cover the end of the digastric notch, the digastric muscle will be felt acting in concert with the omo-hyoid, and the anterior belly can be felt between the jaw and hyoid bone. The peculiar attachment of the digastric can now be appreciated, as the hyoid bone is left sufficiently free in its various movements, although it is at the same time the centre of control and support to the head. The importance of this support to the head can hardly be over-estimated, for the weight of the head beyond the atlas must be balanced. This the digastric and omo-hyoid muscles do effectually by acting upon the jaw, which is a lever whose length below the top of the atlas is over one-third of the height of the head above the atlas. The points from which these muscle act are the mastoid process and the shoulder; the vertex of their angle being in the hyoid bone, from whence they draw in the chin; in this direction they are very active and powerful. They not only balance the head in locomotion and leave the other muscles free to act in deglutition, vocalization, and articulation, but frequently coöperate with them.

(To be Continued.)

## Figures and Tables

**Figure f1:**